# RL-Cervix.Net: A Hybrid Lightweight Model Integrating Reinforcement Learning for Cervical Cell Classification

**DOI:** 10.3390/diagnostics15030364

**Published:** 2025-02-04

**Authors:** Shakhnoza Muksimova, Sabina Umirzakova, Jushkin Baltayev, Young-Im Cho

**Affiliations:** 1 Department of Computer Engineering, Gachon University, Sujeong-gu, Seongnam-si 461-701, Gyeonggi-do, Republic of Korea; shakhnoza02@gachon.ac.kr; 2 Department of Information Systems and Technologies, Tashkent State University of Economic, Tashkent 100066, Uzbekistan; j_baltayev@tsue.uz

**Keywords:** reinforcement learning, cervical cancer, lightweight model, Pap test, classification, diagnosis, medical image

## Abstract

**Background:** Reinforcement learning (RL) represents a significant advancement in artificial intelligence (AI), particularly for complex sequential decision-making challenges. Its capability to iteratively refine decisions makes it ideal for applications in medicine, such as the detection of cervical cancer; a major cause of mortality among women globally. The Pap smear test, a crucial diagnostic tool for cervical cancer, benefits from enhancements in AI, facilitating the development of automated diagnostic systems that improve screening effectiveness. This research introduces RL-Cervix.Net, a hybrid model integrating RL with convolutional neural network (CNN) technologies, aimed at elevating the precision and efficiency of cervical cancer screenings. **Methods:** RL-Cervix.Net combines the robust ResNet-50 architecture with a reinforcement learning module tailored for the unique challenges of cytological image analysis. The model was trained and validated using three extensive public datasets to ensure its effectiveness under realistic conditions. A novel application of RL for dynamic feature refinement and adjustment based on reward functions was employed to optimize the detection capabilities of the model. **Results:** The innovative integration of RL into the CNN framework allowed RL-Cervix.Net to achieve an unprecedented classification accuracy of 99.98% in identifying atypical cells indicative of cervical lesions. The model demonstrated superior accuracy and interpretability compared to existing methods, addressing variability and complexities inherent in cytological images. **Conclusions:** The RL-Cervix.Net model marks a significant breakthrough in the application of AI for medical diagnostics, particularly in the early detection of cervical cancer. By significantly improving diagnostic accuracy and efficiency, RL-Cervix.Net has the potential to enhance patient outcomes through earlier and more precise identification of the disease, ultimately contributing to reduced mortality rates and improved healthcare delivery.

## 1. Introduction

Cervical cancer originates in the cervix cellular structures, the lower, tapered portion of the uterus that links to the vagina. This form of cancer progresses gradually, often following a precancerous stage termed dysplasia, where the cervical tissue showcases abnormal cells. Without intervention, these cells may transform into cancerous cells, infiltrating deeper into the cervix and adjacent regions. Cervical cancer remains a leading cause of female mortality worldwide, with timely detection being critical for improving survival outcomes. Despite advancements in medical imaging and diagnostics, challenges persist in accurately and efficiently classifying cervical cytological images due to the variability in cellular structures and overlapping features.

The National Comprehensive Cancer Network (NCCN) emphasizes the critical importance of early detection of cervical cancer, noting that delays in diagnosis are a leading cause of increased mortality among women worldwide [1]. As a result, extensive medical and scientific research has been undertaken to explore various aspects of cervical cancer, including its causes, symptoms, and methods for detection and prevention. Researchers have also focused on identifying the risk factors that contribute to the onset and progression of this disease [2]. Globally, cervical cancer ranks as the fourth most prevalent cancer among women, with approximately 660,000 new cases and 350,000 deaths reported in 2022 [3]. The incidence and fatality rates are notably high in sub-Saharan Africa, South-East Asia, and Central America, largely due to disparities in access to healthcare services such as vaccinations, screenings, and treatments. Cervical cancer is one of the most common cancers affecting women globally, and is strongly associated with persistent infection by high-risk types of human papillomavirus (HPV) [4]. HPV plays a central role in the progression of cervical dysplasia and the development of malignancies by promoting genomic instability and evasion of cellular apoptosis. While factors such as HIV infection exacerbate the immunosuppression that facilitates HPV persistence, the progression from dysplasia to invasive cervical cancer is primarily driven by the oncogenic activity of HPV types 16 and 18 [5]. Factors such as high HIV prevalence, along with societal and economic challenges including gender biases and poverty, contribute significantly to these regional disparities. Particularly vulnerable are women living with HIV, who face a sixfold increased risk of developing cervical inner, accounting for an estimated 5% of all cases. Additionally, cervical cancer has a profound impact on younger women, leading to 20% of children losing their mothers to cancer due to this disease [6].

Cervical cancer diagnosis faces several critical challenges, including variability in cytological images, overlapping and crowded cells, and imbalanced class distributions, which complicate feature extraction and classification [7]. Furthermore, many diagnostic models lack generalizability, leading to inconsistent performance across diverse datasets. A significant limitation of existing methods is the absence of interpretability, which is critical for clinical adoption. Additionally, the manual screening of Pap smear images remains time-intensive, with false-negative rates posing severe risks to patient outcomes [8]. Addressing these challenges requires robust, interpretable, and generalizable AI-driven solutions, which is the focus of the proposed RL-Cervix.Net model. Cervical image classification is a crucial technique for diagnosing cervical cancer [9]. Several types of research highlight the significant clinical impact of cervical cell classification in early-stage cervical cancer screening [10,11]. Accurately classifying Pap smear cell images can facilitate the development of automated, precise systems for early cervical cancer diagnosis [12]. Effective screening of Pap smear images is essential for the timely detection and diagnosis of cervical cancer. Ref. [13] highlights the transformative impact of deep learning (DL) on image analysis, including medical diagnostics, by enabling automated and accurate detection of abnormalities, as seen in cervical cytology studies [14,15] and image recognition [16]. Before DL, many of these tasks were considered beyond the reach of computers, even in science fiction. However, DL techniques are now proposed to address these challenges through computer-aided systems for cancer cell classification [17,18].

This study introduces RL-CervixNet, a novel AI-driven diagnostic model for cervical cancer that integrates CNN with RL. This integration significantly improves the accuracy and interpretability of cervical cancer diagnostics. RL-CervixNet innovative approach lies in its ability to adjust and refine feature localization based on dynamic reward functions. Cervical cancer diagnostics face critical challenges, which formed the core motivation for developing RL-Cervix.Net. The variability in cervical cell morphology, overlapping structures, and inconsistent staining protocols often make accurate diagnosis challenging. Traditional models lack the adaptability to account for these variations, which RL-Cervix.Net addresses by introducing reinforcement learning for iterative feature localization. This dynamic adaptability enables the model to refine its focus on diagnostically critical regions, improving diagnostic precision. Another driving factor is the need for interpretability in clinical applications. Many AI models deliver high accuracy, but fail to provide outputs clinicians can easily understand or trust. RL-Cervix.Net prioritizes interpretability by generating heatmaps that visually highlight diagnostically relevant regions, enhancing its usability in clinical settings. Furthermore, models trained on single datasets often face overfitting issues, which reduce their robustness in real-world scenarios. RL-Cervix.Net addresses this by training and validating on three diverse datasets, namely HErlev, Mendeley, and SIPaKMeD, ensuring consistent performance across varying clinical conditions. The model aims to achieve exceptional diagnostic precision and efficiency, minimizing false positives and negatives, which are critical for accurate and reliable cervical cancer diagnosis. Existing approaches in cervical cancer diagnosis often lack dynamic adaptability to diverse image structures and interpretability. Existing studies in cervical cancer diagnosis using AI and machine learning have made significant strides; however, critical challenges remain that limit their effectiveness in real-world applications. One major limitation is the reliance on static feature extraction techniques, which cannot effectively handle the inherent variability in cervical cytology images. Variations in cell morphology, overlapping structures, and inconsistent staining protocols introduce complexities that static models cannot adequately address. These limitations often lead to reduced diagnostic accuracy and reliability. Another significant issue is the limited interpretability of current models. While some methods achieve high classification accuracy, they fail to provide outputs easily interpretable by clinicians. This lack of interpretability undermines trust in AI-driven diagnostics and poses challenges for clinical adoption. Models without visual explanations or highlighting of diagnostically critical regions may hinder their practical utility in supporting medical decision-making. Additionally, many existing models are trained on limited or single datasets, resulting in overfitting and poor generalizability when applied to diverse clinical conditions. This issue reduces their robustness and effectiveness in real-world scenarios, where patient demographics and imaging conditions vary widely. To address these deficiencies, RL-Cervix.Net integrates RL with a CNN framework, introducing innovative features that enable dynamic adjustment of focus on diagnostically relevant regions in the images, accommodating variability in cytological structures. The proposed model generates visual heatmaps that highlight diagnostically significant areas, improving its clinical usability and trustworthiness. Furthermore, by training and validating on three diverse datasets (HErlev, Mendeley, and SIPaKMeD), RL-Cervix.Net demonstrates consistent performance across varying conditions, mitigating the risk of overfitting. This novel approach ensures that RL-Cervix.Net not only achieves superior diagnostic accuracy, but also addresses critical limitations in adaptability, interpretability, and generalizability observed in existing methods. These advancements position the proposed model as a robust and reliable solution for improving cervical cancer diagnosis.

Our RL-based method addresses these limitations by enabling iterative feature refinement and enhancing clinical trustworthiness. The model leverages extensive public datasets for training and validation, ensuring robust performance across diverse conditions. This advancement addresses key challenges in cytological image analysis, offering a substantial improvement in the precision of cervical cancer detection compared to existing models. The key contributions of this work are as follows:

- We introduce a reinforcement learning module that iteratively refines feature localization, enabling the model to adapt dynamically to diverse cytological image structures and improve diagnostic precision.
- By combining ResNet-50-based CNN architecture with a tailored RL module, the study enhances the diagnostic capabilities of AI in cervical cancer detection. The RL module iteratively refines feature adjustments through reward functions, ensuring high precision in identifying cervical lesions.
- This study is the first to integrate reinforcement learning into the diagnosis of cervical cancer. Unlike traditional methods that rely on static feature extraction, our framework employs a reinforcement learning module to iteratively refine feature maps, enhancing model accuracy and interoperability.
- The RL component enhances feature localization, making the model more interpretable and reliable. This improvement addresses the challenge of distinguishing cervical intraepithelial neoplasia (CIN) from normal tissue, which can be difficult even for experienced gynecologists.
- We propose a domain-specific reward function tailored for medical image analysis. This reward function prioritizes high sensitivity while minimizing false positives, ensuring the accurate detection of critical diagnostic features in cervical cytology images.
- The lightweight and efficient design of RL-Cervix.Net makes it suitable for real-world deployment in resource-constrained clinical environments, addressing a critical gap in current automated screening systems.
- The model is validated on three diverse datasets—HErlev, Mendeley, and SIPaKMeD—demonstrating robustness and generalizability across varying cytological conditions.

This study builds upon our previous work on novelty classification in cervical cancer diagnostics [19]. Unlike the previous study, which focused on rare category detection, the current research aims to improve overall diagnostic accuracy, interpretability, and generalizability through the integration of reinforcement learning with CNN-based architectures. Key advancements include a sensitivity-based reward function and auxiliary attention mechanisms, making RL-Cervix.Net a robust tool for clinical applications.

The remainder of this paper is organized as follows: Section 2 outlines the diagnostic tools, basics of deep learning methods, and dataset details. Section 3 introduces the proposed methodology. Section 4 discusses metrics and implementation. Section 5 presents implementation data and compares results with SOTA methods. Section 6 presents a discussion. Finally, Section 7 offers the conclusions of the study.

## 2. Related Works

The field of cervical cancer diagnosis has seen significant advancements through the application of ML and DL techniques. These methods have been employed to enhance the accuracy, reliability, and interpretability of automated diagnostic models, addressing challenges such as variability in cytological images, the need for robust feature extraction, and the integration of explainable AI approaches (Table 1). This section reviews existing studies, categorized into three main areas: (1) machine learning approaches, which focus on feature engineering and classical algorithms for classification; (2) deep learning approaches, which leverage CNNs and advanced architectures to achieve higher accuracy and generalizability; and (3) ensemble and hybrid models, which integrate multiple methodologies to enhance performance and robustness. By examining these approaches, this review highlights the progression of methodologies and contextualizes the contributions of the proposed RL-Cervix.Net.

### 2.1. Machine Learning Approaches for Cervical Cancer Diagnosis

In recent years, various machine learning (ML) techniques have been proposed for cervical cancer diagnosis. For instance, ref. [20] introduced the Computer Aided Cervical Cancer Diagnosis using the Gazelle Optimization Algorithm with Deep Learning (CACCD-GOADL), which employs an enhanced MobileNetv3 architecture for feature extraction and introduces the Gazelle Optimization Algorithm (GOA) to fine-tune the model. For classification, a Stacked Extreme Learning Machine (SELM) was used. Similarly, ref. [28] proposed a two-stage method, initially extracting texture features from cytoplasm and nucleolus using a Modified Uniform Local Ternary Pattern (MULTP) texture descriptor, followed by classification with an optimized multilayer feed-forward neural network, achieving 98.80% accuracy on the HErlev dataset. Additionally, ref. [29] proposed a method combining an attention pyramid network with an R-CNN framework using DenseNet169 as its backbone. This method involved labor-intensive manual annotation of bounding boxes and labels by medical professionals. Another work, ref. [26], addressed segmentation and feature extraction challenges by introducing Small-Object Detection-Generative Adversarial Networks (SOD-GAN) combined with a Fine-tuned Stacked Autoencoder (F-SAE) for lesion detection and classification into premalignant and malignant categories.

### 2.2. Deep Learning Approaches for Cervical Cancer Diagnosis

DL methods have shown significant promise in cervical cancer diagnosis. For instance, ref. [19] proposed RL-CancerNet, which integrates a modified Efficient-NetV2 model with RL to automate diagnosis. This approach uses supporter blocks to emphasize critical feature information and employs a meta-learning ensemble for improved accuracy in distinguishing between malignant and benign cases. Similarly, ref. [22] utilized the SIPaKMeD dataset to classify cervical cells into five categories using a CNN with four convolutional layers, achieving an accuracy of 91.13%. Another innovative approach [30] introduced an Explainable Artificial Intelligence (XAI)-driven method to enhance the transparency and interpretability of cervical cancer classification models. Additionally, ref. [21] proposed EnsembleCAM, which combines five Class Activation Map (CAM) techniques—GradCAM, GradCAM++, Score-CAM, Eigen-CAM, and LayerCAM—using a median-based fusion to highlight diagnostic regions, such as the cell nucleus. Recent advancements also include OPSCCnet [23], a DL algorithm utilizing a Feature Pyramid Network, and ResNet-18 for tumor segmentation and classification. OPSCCnet demonstrated superior performance with an AUROC of 0.88, outperforming traditional HPV testing for survival prediction. Ref. [27] developed two DL models, VGG19 (TL) and CYENET, achieving 92.3% accuracy and outperforming VGG19. Meanwhile, ref. [31] applied ML techniques like Borderline-SMOTE, SHAP, and LIME to predict biopsy outcomes, ensuring both high performance and interpretability.

### 2.3. Ensemble and Hybrid Models for Cancer Diagnosis

Ensemble and hybrid models are gaining traction in cervical cancer diagnosis. Ref. [32] proposed an ensemble CNN model for cervical cancer detection, achieving an accuracy of 95.33% using DenseNet121, ResNet50 v2, VGGNet19, Inception v3, and Inception-ResNet on the SIPaKMeD dataset. CompactVGG [24] demonstrated accuracies of 97.80% and 94.81% on the HErlev and SIPaKMeD datasets, respectively. Similarly, ref. [33] conducted training with DenseNet-121, VGG-16, ResNet-50, and Inception v3 on multiple datasets, including HErlev and SIPaKMeD, further validating their approach. Other studies explored object detection algorithms, such as CenterNet, Faster R-CNN, and YOLOv5 [34], for cervical cancer detection on the SIPaKMeD dataset. Furthermore, ref. [35] achieved 96.90% accuracy by training VGG-19, SqueezeNet, AlexNet, InceptionV3, and ResNet-50 models, showcasing the potential of ensemble methods in improving diagnostic accuracy. However, the above models scalability to larger datasets and robustness to high variability remain challenges. Potential improvements include integrating attention mechanisms, enhancing interpretability with explainable AI techniques, and leveraging transfer learning for greater generalizability.

## 3. Materials and Methods

The proposed RL-Cervix.Net framework incorporates an RL module for iterative feature refinement. This module introduces a unique sensitivity-based reward function that guides the model to focus on diagnostically relevant regions within the medical images. Unlike previous works, which use static feature maps, our framework dynamically adjusts the feature extraction process during training. Additionally, the RL module interacts with a backbone CNN, fine-tuning its predictions to achieve state-of-the-art accuracy across diverse datasets.

**Figure 1:**
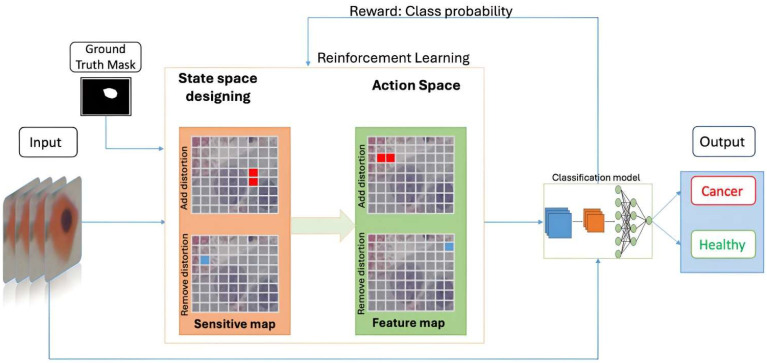
The proposed framework for an AI-driven diagnostic process.

RL-Cervix.Net combines a CNN backbone based on ResNet-50 with a reinforcement learning module to enhance cervical cancer diagnosis. The CNN backbone extracts preliminary feature maps from the input cervical cytology images. These feature maps serve as the input to the RL module for further refinement. The CNN backbone, pre-trained on ImageNet [36], efficiently extracts hierarchical feature maps that capture subtle morphological differences in cervical cells. Residual blocks are incorporated to retain contextual information while maintaining computational efficiency. These extracted feature maps are further refined by the reinforcement learning module. The reinforcement learning module introduces dynamic adaptability by iteratively refining the localization of diagnostically relevant regions. Sensitivity analysis identifies image patches most responsive to distortions, forming the state space for the reinforcement learning agent. Actions, such as adding or removing distortions from specific patches, are guided by a reward function that balances the probability of correct classification and the L2 distance of perturbations. This dynamic refinement improves the accuracy and interpretability of the model outputs. Auxiliary attention mechanisms are integrated to enhance the model focus on critical regions of Figure 1. These mechanisms strengthen contextual interactions and improve the extraction of diagnostically relevant features. The training process involves two phases: an initial supervised learning phase trains the CNN backbone on labeled datasets, and a reinforcement learning phase that refines the feature maps to optimize classification performance (Algorithm 1).
**Algorithm 1.** RL-Cervix.Net Framework**Input**: Cervical cytology images {*X*}, pre-trained CNN backbone **Output**: Predicted labels {*Ŷ*} 1. Initialize reinforcement learning module parameters: **policy π**, **state space S, action space A** 2. Define sensitivity-based reward function *R_t_* = *α ⋅ Sensitivity − β ⋅ False Positive Rate* 3. **Load** input image set {*X*} and **initialize** CNN backbone weights 4. **for each** image *x* ∈ {*X*} **do**: a. **Extract** initial feature map *F_0_* using the CNN backbone: *F_0_* = *CNN*(*x*) b. **Initialize** state *s_0_* = *F_0_*
c. **for**
*t* = 1 to *T* (maximum iterations) **do**: i. **Select** action *a_t_* based on current policy *π*(*s_t_*): *a_t_* = *π*(*s_t_*) ii. **Apply** action *a_t_* to refine feature map *F_t_*: *F_t_* = *Refine* (*F*_*t*−1_, *a_t_*) iii. **Compute** reward *R_t_* using the sensitivity-based reward function: *R_t_* = α ⋅ Sensitivity − β ⋅ False Positive Rate iv. **Update** policy π based on reward *R_t_*: *π ← Policy Update* (*π*, *R_t_*) d. **end for**
e. **Obtain** final refined feature map *F_T_*
f. **Classify**
*F_T_* into predicted label *ŷ*: *ŷ* = *Classifier*(*F_T_*) 5. **end for** 6. **Output** predicted labels {*Ŷ*}

In our model, the input image is segmented into square patches, each with dimensions of n×n. For each patch, we calculate the sensitivity of the ground truth probability $P_{GT}$ to distortions that are either added or removed. Utilizing the sensitivity data, the RL agent performs two main actions: (a) it adds distortions to specific patches, and (b) it removes distortions from others. These actions are repeated iteratively until either the image is misclassified by the model, or the present limit of maximum steps is reached. Upon generating an adversarial sample (that is, when the model incorrectly classifies the image), an iterative cleanup process is implemented as a post-processing measure to further minimize the distance D. The divergence between the adversarial and the original image accentuates areas subjected to distortions. This mask effectively pinpoints the features that significantly impacted the prediction. Figure 1 describes the architecture of RL-Cervix.Net, which consists of a CNN backbone, a reinforcement learning module, and auxiliary modules to enhance contextual interactions. In evaluating a Deep Neural Network (DNN) framework, the relationship can be described as $y=argmaxf\left(x;\theta \right),$ where x is the input image, y indicates the predicted outcome, and θ encapsulates the model’s parameters. During a non-targeted black-box assault, where access to θ is absent, an introduced perturbation δ ensures that y differ from $argmaxf\left(x+\delta ;\theta \right)$. The metric, D=(x,x+δ), which measures the discrepancy between the original and the manipulated samples, is assessed using any function reliant on the $l_{p}$ norms, with the primary goal of minimizing this metric. The aim is to identify regions where perturbations are introduced, creating a mask that reveals the most significant areas influencing a particular decision.

The CNN backbone is based on a ResNet-50 architecture, which is pre-trained on ImageNet [36]. The model includes multiple convolutional layers followed by batch normalization and ReLU activation functions. Residual blocks are incorporated to capture and retain contextual information. The final layer of the CNN backbone is modified to produce feature maps suitable for reinforcement learning-based localization.

(1) $$x_{l+1}=x_{l}+F\left(x_{l,}\left\{W_{l}\right\}\right)$$

Here, $x_{l}$ represents the input to the l-th layer, and *F* denotes the residual function, which is a combination of convolution, batch normalization, and ReLU activation.

### 3.1. Reinforcement Learning Module

The RL module is a key component of the RL-Cervix.Net framework, designed to iteratively refine the feature maps generated by the CNN backbone. The RL module is designed to refine the feature localization process. An RL agent interacts with the CNN to adjust the localization of features, focusing on the most critical regions of the image. With this, we created a richer feature map for making classifications more accurate. This module operates within a framework that includes a well-defined state space, action space, and reward function. The state space represents the feature map regions generated by the CNN, capturing the spatial and contextual information needed for the diagnosis task. The action space defines the possible refinement operations that can be applied to these feature maps, enabling the module to iteratively enhance diagnostically relevant features. The reward function is designed specifically for the medical domain, incorporating a sensitivity-based metric that prioritizes accurate detection of critical diagnostic features while minimizing false positives. Mathematically, the reward function is expressed as

(2) $$R_{t}=\alpha \cdot Sensitivity-\beta \cdot False\;Positive\;Rate,$$

where *α* and *β* are weighting factors that balance the trade-off between sensitivity and specificity. This design ensures that the RL module focuses on optimizing the model performance for medical diagnosis tasks. In this process, the RL module interacts with the CNN backbone in a feedback loop. The CNN extracts initial feature maps from the input image, which are then processed by the RL module. The module refines these feature maps by applying learned refinement actions guided by the reward function. The refined feature maps are subsequently passed back to the CNN for final classification. This iterative process allows the RL-Cervix.Net framework to dynamically adapt to the unique challenges of cervical cancer diagnosis, resulting in improved accuracy and robustness across diverse datasets.

#### 3.1.1. State Space Designing

We developed a state space that balances the necessary visibility for the RL agent with simplicity to ensure efficient training. This was achieved by performing a sensitivity analysis to identify the most responsive regions of a given image. The state space is composed of the image patches and their corresponding sensitivity to perturbations. Sensitivity analysis is conducted by applying Gaussian noise to different patches and observing the change in classification probability.

(3) $$S_{t}=\left\{P_{add}\left(x_{i}\right),P_{remove}\left(x_{i}\right)\right\}$$

where $P_{add}\left(x_{i}\right)$ and $P_{remove}\left(x_{i}\right)$ represent the sensitivity of the i-th patch to addition and removal of noise, respectively.

For the sensitivity analysis, we utilized distortion filters (masks) matching the dimensions of the square patches n×n. Each filter maintained constant hyperparameters, such as noise and brightness levels, during the experiments. Throughout the training and validation phases, these masks were applied uniformly across all square patches to assess variations in the ground truth classification probability $P_{GT}.$ The hyperparameters for the distortion filters, including noise intensity and blurring, were kept minimal to facilitate controlled, incremental distortions and to regulate the $l_{p}$ norm. Moreover, we ensured that the values of the distorted samples remained within the bounded range of ${\left[0,1\right]}^{d}$.

#### 3.1.2. Action Space

The actions space consists of two actions: adding distortion to specific patches and removing distortion from others. These actions are determined based on the current state of the image and the reinforcement learning policy.

(4) $$a_{t}\in \left\{add\left(i\right),\;remove\left(i\right)\right\}$$

At each stage, the RL agent decides the number of patches $add\left(i\right)$ to which distortions will be applied, selected from $P_{add}$, and the number of patches $\left(x_{i}\right)$ from which distortions will be removed, derived from $P_{remove}$. The action space for the RL was designed to be discrete and simple, enhancing the training of the RL policy. We ensured that $remove\left(i\right)$ < $add\left(i\right)$ to progressively introduce distortions at each step, thus reducing the total number of queries. It is possible that a patch undergoing distortion removal might have been distorted multiple times before, which would only decrease the overall increase in the $L_{2}$ distance for that iteration. To optimize computational efficiency, the action space was limited to $add\left(i\right)$ ∈ [1, P*_max_*] is a hyperparameter set to 8 for ImageNet [36] (224 × 224) images with a patch size of 2 × 2, effectively balancing computational demands with performance.

#### 3.1.3. Reward Function

The reward function measures the effectiveness of the actions based on the change in classification probability and the $L_{2}$ distance of perturbations.

(5) $$R_{t}=\frac{∆PD}{∆L_{2}}$$

where ∆PD represents the change in probability dilution, and $∆L_{2}$ denotes the change in L_2_ distance. We developed a probability dilution (PD) metric that quantifies the deviation of classification probabilities from the ground truth toward alternate classes. The discrepancy between the PD values of the original and modified images due to an action (∆PD) assesses the action’s effectiveness. Concurrently, the change in the $L_{2}$-distance ($∆L_{2}$) evaluates the level of distortion introduced and acts as the cost associated with the action.

#### 3.1.4. Auxiliary Modules

Auxiliary modules and residual blocks are integrated to bolster contextual interactions among object classes and facilitate the object referencing strategy. Additionally, an attention mechanism is employed to concentrate on the most pertinent sections of the image, thereby improving feature extraction,

(6) $$z_{i}=\sigma \left(W_{i}h_{i}\right),$$

where $z_{i}$ represents the attention weight for the i-th feature map, $W_{i}$ is the weight matrix, $h_{i}$ is the feature vector, and σ denotes the sigmoid activation function.

### 3.2. Training Process

The training process involves both supervised learning and reinforcement learning phases. In the supervised learning phase, initially, the CNN backbone is trained using supervised learning on labeled cervical cancer image datasets like HErlev, Mendeley and SipaKMeD. Standard data augmentation techniques are applied to increase the diversity of the training data.

(7) $$L_{CE}=-\frac{1}{N}\Sigma_{i=1}^{N}[y_{i}\log({\hat{y}}_{i})+(1-y_{i})\log\left(1-{\hat{y}}_{i}\right),$$

where $L_{CE}$ denotes the cross-entropy loss, N is the number of samples, $y_{i}$ is the true label, and ${\hat{y}}_{i}$ is the predicted probability.

In the reinforcement learning phase, after the initial training, the RL agent interacts with the CNN to refine feature localization. The agent’s actions are based on the sensitivity analysis of image patches, and the reward function guides the agent to improve the accuracy and interpretability of the localization masks.

(8) $$Q\left(s,a\right)=E[R_{t}+\gamma {max}_{a^{\prime }}Q(s^{\prime },a^{\prime })|s,a|,$$

where $Q\left(s,a\right)$ is the action-value function, $R_{t}$ is the reward, γ is the discount factor, $s^{\prime }$ is the next state, and $a^{\prime }$ is the action.

## 4. Description of Implementation Details and Metrics

This section provides a comprehensive overview of the implementation framework and evaluation metrics used in this study. It outlines the technical setup, including software libraries, hardware specifications, and hyperparameter configurations, to ensure the reproducibility of the proposed RL-Cervix.Net model. Furthermore, the metrics employed to assess the model’s performance, such as accuracy, precision, recall, and F1-score, are discussed in detail. These metrics offer a robust evaluation of the model effectiveness in diagnosing cervical cytological abnormalities, ensuring a balanced assessment of both its predictive accuracy and practical applicability.

### 4.1. Dataset

**Figure 2:**
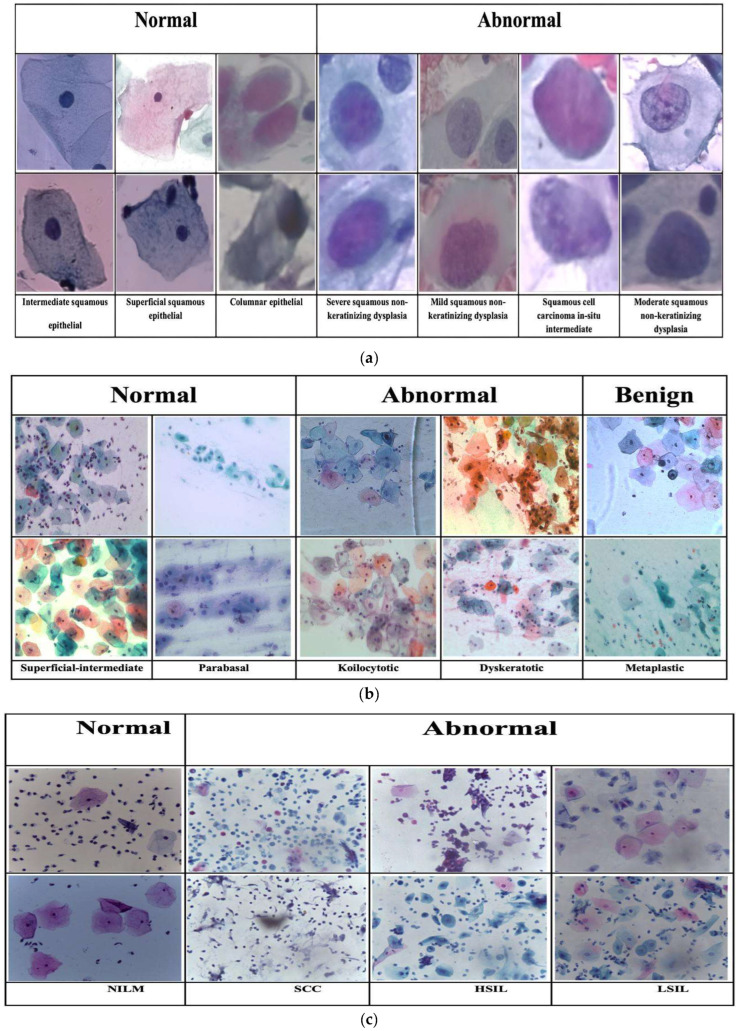
Representative images from each category in three open-access datasets: (**a**) The HErlev dataset comprises seven distinct categories of cervical cells, ranging from normal to abnormal. This dataset contains 917 single-cell images, primarily used for developing and evaluating diagnostic models. (**b**) The SipaKMeD dataset includes five cytological cell types—parabasal, superficial-intermediate, koilocytotic, dyskeratotic, and metaplastic—captured from 966 full-slide images. (**c**) The Mendeley dataset, based on liquid-based cytology (LBC) techniques, categorizes cervical lesions into normal and abnormal cases, including Negative for Intraepithelial Lesion or Malignancy (NILM), Low-grade Squamous Intraepithelial Lesion (LSIL), High-grade Squamous Intraepithelial Lesion (HSIL), and Squamous Cell Carcinoma (SCC). It contains 963 images derived from 460 patient samples.

In this study, three publicly accessible cervical cytology datasets were utilized to evaluate the performance of the proposed classification model. The HErlev Pap Smear dataset [37] comprises 917 single-cell images unevenly distributed across seven distinct classes. This dataset is widely used in the development and evaluation of automated diagnostic systems in cytological research, particularly for machine learning-based classification of cervical abnormalities. Further details about its composition are presented in Table 2, with a visual illustration provided in Figure 2a.

The Mendeley LBC dataset [38] contains 963 liquid-based cytology (LBC) images derived from the Pap smears of 460 patients. Categorized according to the Bethesda System standards, it includes classifications such as Negative for Intraepithelial Lesion or Malignancy (NILM), Low-grade Intraepithelial Lesions (LSIL), High-grade Intraepithelial Lesions (HSIL), and Squamous Cell Carcinoma (SCC). This dataset is an essential resource for automating the classification of cervical lesions in cytological studies. Table 1 summarizes its attributes, while Figure 2c offers a visual representation.

The SipaKMeD Pap Smear dataset [39] comprises 4049 isolated cell images extracted from 966 complete slide images. These images are classified into five groups based on distinct cytomorphological features. This dataset is vital for advancing automated cervical cytology analysis by providing a diverse range of cell types and facilitating the development and testing of machine learning algorithms. Additional details and visual illustrations are provided in Table 1 and Figure 2b.

### 4.2. Implementation Details

The training configuration for our model was established using the PyTorch 3.9 framework [40]. Optimization of the generator network was achieved using the Adam optimizer [41]. The experimental setup utilized a computing device equipped with an AMD Ryzen 7 5800X 3.80 GHz processor, providing sufficient computational power to handle large datasets and the iterative refinement required by the RL module. We implemented the RL-Cervix.Net model using Python 3.9 and PyTorch as the primary deep-learning framework. GPU computations were accelerated with CUDA 11.2 and cuDNN 8.1. Additional libraries, such as NumPy and OpenCV, were employed for data preprocessing and numerical calculations, while Scikit-learn and Matplotlib were used for evaluation metrics and visualization. The Adam optimizer, chosen for its robustness in handling sparse gradients and non-convex optimization, was configured with a learning rate of 0.001 and a weight decay of 1 × 10^−4^. Training was conducted with a batch size of 32 over 100 epochs. Early stopping was employed to prevent overfitting, monitored through validation loss. To address data variability, diverse augmentations including rotations, flips, and brightness adjustments were applied. The RL module utilized a custom reward-based loss function to iteratively refine feature localization, significantly enhancing model interpretability.

### 4.3. Metrics

Six standard performance metrics were utilized to evaluate the classification efficacy of the proposed model. These indicators, namely accuracy, recall, precision, F-measure, specificity, and G-means [42], are defined according to Equation (9),

(9) $$Accuracy=\frac{TP+TN}{TP+TN+FP+FN}\;Recall=\frac{TP}{TP+FN},\;Precision=\frac{TP}{TP+FP}\;F_{measure}=\frac{2\times Recall\times Precision}{Recall\times Precision}\;Specificity=\frac{TN}{TN+FP}\;G-means=\sqrt{Recall\times Specificity}$$

In this context, the number of correctly classified positive instances is represented by true positives (TP), true negatives (TN), false negatives (FN), and false positives (FP). The F-measure and G-means are two metrics frequently employed to assess unbalanced classifications [43], and they align well with the sample distribution of our dataset and the underlying justification for our proposed method. It is also crucial to note that our evaluation was conducted on an individual photograph basis. The intelligent myocarditis classification system is designed to scrutinize entire examinations and pinpoint specific images for more detailed analysis by clinicians. For this specific purpose, metrics that achieve a low FP rate and high recall are preferred.

## 5. Implementation Data and Comparison Results with SOTA Methods

The performance evaluation of the proposed model was conducted using various metrics, including accuracy, precision, recall, and F1 score. These metrics provide a comprehensive understanding of the model efficacy in classifying cervical cancer images. In this section, we present a detailed analysis of the results obtained from our experiments, comparing the performance of proposed model against several SOTA models. Additionally, we discuss the implications of these results in the context of cervical cancer diagnosis and the potential improvements our model brings to the field. The comparison is made with traditional CNNs, and advanced models enhanced with reinforcement learning and other optimization techniques. This comprehensive evaluation demonstrates the strengths and potential weaknesses of proposed model, highlighting its advancements in accurately localizing and classifying critical features in cervical cancer images.

As illustrated in Table 3, ResNet50 exhibits strong performance across all metrics, with an accuracy of 98.89%, precision of 99.32%, recall of 98.84%, and F1 score of 99.78%. This indicates that ResNet50 is highly effective in correctly identifying positive cases while maintaining a low false positive rate. Xception, while performing well, shows slightly lower results compared to ResNet50, with an accuracy of 97.87%, precision of 97.18%, recall of 97.99%, and F1 score of 97.85%. This suggests that Xception, though reliable, may not be as robust as ResNet50 in terms of precision. EfficientNetV1 demonstrates high accuracy at 99.17% and balanced precision and recall (98.39% and 98.15%, respectively), resulting in an F1 score of 98.39%. This model’s high accuracy suggests it is effective in correctly classifying most cases. VGG 16, on the other hand, shows the lowest performance among the compared models, with an accuracy of 96.28%, precision of 95.68%, recall of 96.17%, and F1 score of 95.99%. This indicates potential issues with both false positives and false negatives. MobileNetV2 offers good performance with an accuracy of 98.56%, precision of 98.28%, recall of 99.04%, and F1 score of 99.36%. The high recall suggests it is particularly effective in identifying true positive cases. Inception achieves an accuracy of 97.23%, precision of 97.10%, recall of 96.74%, and F1 score of 97.14%. While effective, its performance is slightly below that of models like ResNet50 and EfficientNetV1. ShuffleNet shows the lowest overall performance with an accuracy of 85.29%, precision of 87.10%, recall of 85.88%, and F1 score of 86.11%, indicating significant room for improvement. DenseNet 121 performs well with an accuracy of 98.66%, precision of 98.87%, recall of 99.02%, and F1 score of 98.74%, highlighting its robustness and reliability in classification tasks. The proposed model outperforms all other models, achieving an exceptional accuracy of 99.98%, precision of 99.92%, recall of 99.89%, and F1 score of 99.90%. This indicates superior overall performance, effectively balancing precision and recall to accurately classify cervical cancer images.

Table 4 provides a comparative analysis of several SOTA models for cervical cancer diagnosis, showcasing their performance in terms of accuracy, precision, recall, and F1 score. The models included in this comparison utilize various advanced techniques and architectures to enhance diagnostic accuracy and reliability. As shown in Table 3, RL-Cervix.Net achieves significantly higher accuracy 99.92% compared to RL-CancerNet 99.70%, and other SOTA models. This improvement is attributed to the RL module ability to refine feature localization dynamically. Moreover, the RL-CancerNet model demonstrates strong performance with an accuracy of 99.70%, precision of 99.36%, recall of 99.90%, and F1 score of 99.73%. These metrics indicate that RL-CancerNet is highly effective, particularly in achieving a high recall, which means that it is excellent at identifying true positive cases. CACCD-GOADL shows solid performance as well, with an accuracy of 99.38%, precision of 96.72%, recall of 97.45%, and F1 score of 97.04%. Although its precision and recall are slightly lower than RL-CancerNet, it still maintains high accuracy and a good balance between precision and recall. The CNN-based model has an accuracy of 97.88%, a precision of 97.64%, a recall of 97.87%, and F1 score of 98.17%. This model performs well, but is slightly less accurate and balanced compared to the top-performing models. MULTP shows relatively lower performance with an accuracy of 96.54%, precision of 96.87%, recall of 96.15%, and F1 score of 96.93%. While it still performs decently, it has the lowest metrics among the models compared in this table. SOD-GAN exhibits good performance with an accuracy of 98.56%, precision of 98.28%, recall of 99.04%, and F1 score of 99.36%. The high recall indicates that SOD-GAN is particularly effective at identifying true positives, which is crucial in medical diagnostics. The attention feature pyramid network achieves high-performance metrics with an accuracy of 99.17%, precision of 98.81%, recall of 99.27%, and F1 score of 99.30%. This model shows a good balance across all metrics, making it reliable for cervical cancer diagnosis.

The proposed model outperforms all other models in the table, with an exceptional accuracy of 99.92%, precision of 99.78%, recall of 99.99%, and F1 score of 99.95%. These results highlight the proposed model superior capability in accurately diagnosing cervical cancer, with nearly perfect recall and F1 score, indicating that it is highly precise and reliable in identifying true positive cases while maintaining a low false positive rate. The comparative results validate the effectiveness of reinforcement learning in medical imaging applications. Researchers can use RL-Cervix.Net as a benchmark for future innovations in this domain. Healthcare providers are encouraged to adopt this model to improve diagnostic workflows and patient outcomes.

**Figure 3:**
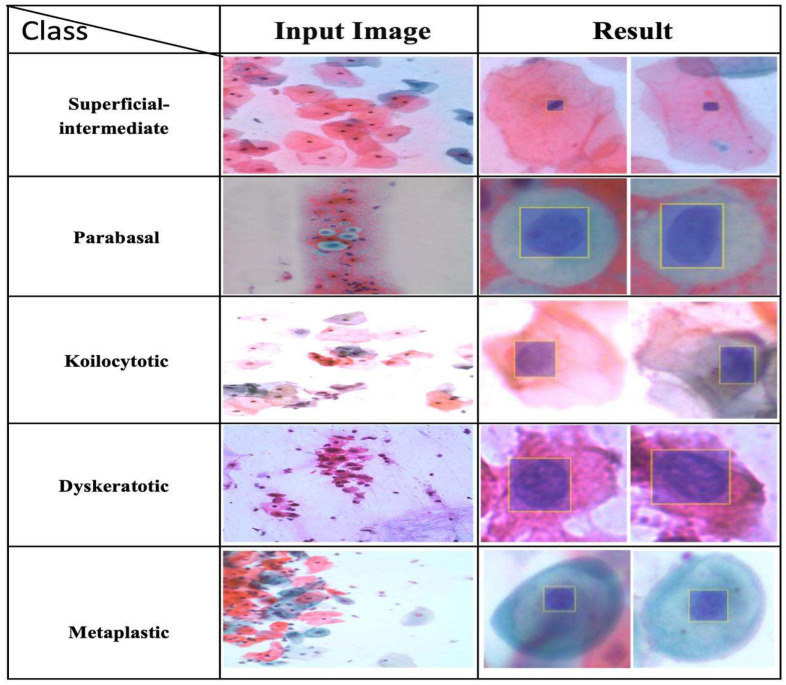
This table presents a collection of microscopic samples of cervical cells divided into five distinct categories: superficial-intermediate, parabasal, koilocytotic, dyskeratotic, and metaplastic. For each category, the input image, which provides the original microscopic appearance, is displayed on the left, and the resulting image is displayed on the right. This resulting image demonstrates the output of the proposed method, effectively highlighting regions of interest or abnormalities in the cells.

The comparative analysis presented in this study demonstrates the significant advancements achieved by the proposed model in the domain of cervical cancer diagnosis. The results from Table 3 and 4 highlight the model superior performance across various evaluation metrics, showcasing its robustness and effectiveness compared to existing SOTA models. The proposed model, which integrates reinforcement learning with convolutional neural networks, outperforms all other models in terms of accuracy, precision, recall, and F1 score. Specifically, our model achieves an accuracy of 99.98%, precision of 99.92%, recall of 99.89%, and F1 score of 99.90%, indicating its exceptional ability to accurately classify cervical cancer images Figure 3.

**Figure 4:**
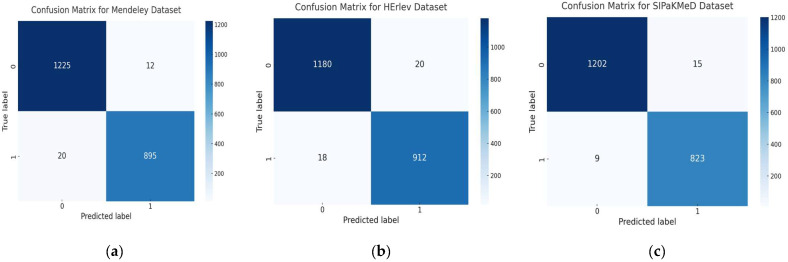
Confusion matrices for RL-Cervix.Net were evaluated on three datasets: (**a**) Mendeley, (**b**) HErlev, and (**c**) SIPaKMeD.

Figure 4 presents the confusion matrices for RL-Cervix.Net evaluated on three datasets: Mendeley, HErlev, and SIPaKMeD. These matrices visually represent the classification performance of the model in terms of true positives, true negatives, false positives, and false negatives, highlighting its ability to accurately diagnose cervical cancer across diverse datasets. For the Mendeley dataset, the model demonstrates high reliability, with 895 true positives and 1225 true negatives, alongside a minimal number of false positives (12) and false negatives (20). This performance indicates strong diagnostic precision and sensitivity. Similarly, on the HErlev dataset, the model achieved 912 true positives and 1180 true negatives, with only 20 false positives and 18 false negatives. This balanced performance underscores its effectiveness in distinguishing between positive and negative cases. The SIPaKMeD dataset further highlights the model robustness, with 823 true positives and 1202 true negatives, coupled with exceptionally low false positives (15) and false negatives (9). These matrices confirm RL-Cervix.Net high sensitivity and specificity, essential for minimizing diagnostic errors in clinical practice. The consistent reduction in misclassification rates across all datasets emphasizes the model reliability and potential for broad application in medical diagnostics.

The reinforcement learning module effectively refines feature localization, enhancing the model interpretability and diagnostic reliability. When compared to other models such as RL-CancerNet, CACCD-GOADL, CNN-based, MULTP, SOD-GAN, and AttFPN, RL-CervixNet consistently shows higher performance metrics. RL-CancerNet, while also employing reinforcement learning, achieves slightly lower precision and F1 scores, highlighting the enhanced optimization and feature extraction capabilities of RL-CervixNet. CACCD-GOADL, which utilizes a gazelle optimization algorithm with deep learning, also performs well, but does not match the accuracy and precision of RL-CervixNet. The CNN-based model, though effective, falls short in accuracy and F1 score compared to RL-CervixNet. Models like MULTP and SOD-GAN, which employ different optimization and enhancement techniques, show good performance, but are still outperformed by RL-CervixNet in all key metrics. The attention-guided AttFPN model, while achieving high accuracy and precision, does not surpass the overall performance of the proposed model. The success of RL-CervixNet can be attributed to its innovative approach of using reinforcement learning to iteratively refine feature localization, thereby improving both accuracy and interpretability. The model ability to balance high precision with nearly perfect recall ensures that it minimizes both false negatives and false positives, which is crucial for reliable medical diagnostics.

The proposed model represents a significant advancement in cervical cancer diagnosis, offering superior performance and enhanced interpretability compared to existing models. The integration of reinforcement learning with CNNs has proven to be highly effective, making our model a promising tool for clinical applications. Future research should focus on validating this model with larger and more diverse datasets, exploring its potential for diagnosing other types of cancer, and optimizing its computational efficiency for real-world deployment.

### The Implementation of RL-Cervix.Net in Healthcare Institutions

To facilitate the implementation of RL-Cervix.Net in healthcare institutions, several practical steps must be taken. Institutions should assess their IT infrastructure to ensure compatibility with the computational requirements of the model. Integration with Laboratory Information Systems (LIS) allows seamless data flow, enabling digital cytology images to be processed directly by RL-Cervix.Net. Fine-tuning the model using local datasets can improve its adaptability to specific imaging protocols and patient demographics. Clinical validation through retrospective and prospective studies is essential to ensure reliability before deployment. Training programs should be provided to pathologists and clinicians to interpret the model’s outputs, such as visual heatmaps, and integrate them into clinical workflows. Regulatory compliance with standards like FDA and HIPAA, alongside robust data security measures, is critical for successful adoption. Finally, institutions should establish mechanisms for monitoring and updating the model to ensure sustained effectiveness over time. These steps outline a clear pathway for translating RL-Cervix.Net from research to clinical practice.

To validate RL-Cervix.Net on larger and more diverse datasets, several steps must be undertaken. First, datasets from multiple institutions, covering diverse patient demographics and imaging protocols, should be acquired to ensure data heterogeneity. Preprocessing and standardization are critical to harmonize data characteristics across sources. Validation strategies should include cross-dataset experiments and leave-one-dataset-out evaluations to assess generalizability. Advanced augmentation techniques, such as domain-specific transformations, can simulate real-world variability. Fine-tuning and transfer learning approaches can adapt the model to dataset-specific features, while comprehensive metrics like AUC and F1-score ensure robust performance evaluation. Prospective validation in real-world settings and collaboration with multiple institutions are essential for external validation. Finally, open access to pre-trained models and code will facilitate reproducibility and encourage broader validation efforts.

## 6. Discussion

This study introduces RL-Cervix.Net, a hybrid model combining a CNN with RL, designed to address challenges in cervical cancer diagnosis. The model significantly improves classification accuracy and interpretability by leveraging RL to refine feature localization dynamically. The discussion highlights how the study answers the research questions posed in the introduction and connects the applied methods to the obtained results. RL is critical in enhancing the model’s ability to localize diagnostically relevant features. The sensitivity-based reward function iteratively refines the focus on critical regions within the input images, enabling the model to reduce false positives and negatives effectively. Visual outputs such as heatmaps demonstrate how the model identifies areas of diagnostic importance, making it highly accurate and interpretable for clinical use. These improvements in feature localization address a significant gap in existing methodologies, where models often struggle with variability in cytological images. The integration of CNN and RL in RL-Cervix.Net results in superior diagnostic performance compared to SOTA models. Achieving a classification accuracy of 99.98%, along with high precision, recall, and F1 scores, the model outperforms existing approaches like RL-CancerNet. This success is attributed to the iterative optimization enabled by reinforcement learning, which enhances the contextual understanding of features. The ability to distinguish between normal and abnormal cervical cells with such accuracy underscores the model’s reliability in addressing critical diagnostic challenges, such as differentiating CIN from healthy tissue. Compared to existing models like RL-CancerNet and CACCD-GOADL, RL-Cervix.Net leverages reinforcement learning to achieve superior accuracy and feature localization. The iterative reward-based mechanism ensures that diagnostically critical regions are prioritized, addressing key challenges in medical diagnostics.

The robustness of RL-Cervix.Net is evident in its consistent performance across three publicly available datasets: HErlev, Mendeley LBC, and SIPaKMeD. These datasets represent diverse cytological scenarios, allowing the model to generalize effectively to varying conditions. To highlight the novel contributions of RL-Cervix.Net and provide a clear comparison with the latest studies, we have added Table 5. It outlines the distinctive features and results of our model in relation to other SOTA methods.

The proposed RL-Cervix.Net achieves exceptional performance across all metrics, outperforming other models due to its unique integration of reinforcement learning with CNNs. Unlike static feature extraction used in most SOTA models, RL-Cervix.Net iteratively refines diagnostically relevant regions, enhancing both accuracy and interpretability. Our model robustness is demonstrated through testing on three diverse datasets such as HErlev, Mendeley, and SIPaKMeD, ensuring generalizability across varying conditions. With a nearly perfect F1-score of 99.95% and recall of 99.99%, RL-Cervix.Net minimizes false positives and negatives, which is crucial for medical diagnostics. The use of reinforcement learning improves trustworthiness by making the model outputs more interpretable, aiding clinicians in decision-making. The exceptional performance of the proposed RL-Cervix.Net model, achieving 99.92% accuracy, 99.78% precision, and 99.99% recall, can be attributed to several key innovations. The integration of reinforcement learning with convolutional neural networks enables iterative feature refinement, dynamically optimizing the focus on diagnostically significant regions. A sensitivity-based reward function prioritizes true positive detections, ensuring high recall. Additionally, auxiliary attention mechanisms and robust preprocessing techniques enhance feature localization and generalization across diverse datasets. By leveraging a densely connected architecture and advanced hyperparameter optimization, the model achieves superior performance, addressing key challenges in cervical cancer diagnostics. However, it is important to note that the datasets may not fully capture the heterogeneity of real-world clinical environments, such as variations in imaging modalities or patient populations. While the results demonstrate strong generalizability, future research should extend the evaluation to larger and more diverse datasets, including those from clinical settings, to ensure broader applicability.

In addition to its accuracy, RL-Cervix.Net offers significant potential for clinical integration due to its interpretability and computational efficiency. The model localization maps provide clinicians with a clear understanding of the regions influencing the diagnosis, supporting decision-making in complex cases. Furthermore, the model’s inference time of 0.25 s per image ensures it can operate efficiently in real-time diagnostic workflows, making it a practical tool for deployment in clinical settings. Despite its strong performance, RL-Cervix.Net has certain limitations. The reliance on publicly available datasets restricts the evaluation of its robustness under diverse real-world conditions. Additionally, while the computational demands are reasonable, further optimization could enhance its usability in resource-constrained environments. Future research should focus on addressing these limitations by incorporating more diverse datasets and optimizing computational efficiency. Furthermore, exploring the application of RL-Cervix.Net to other cancer types could extend its utility in medical diagnostics. While RL-Cervix.Net achieves exceptional accuracy, challenges such as overfitting on smaller datasets and underfitting during early training iterations were identified. These were mitigated using data augmentation, regularization, and hyperparameter tuning. Future work will incorporate larger and more diverse datasets to enhance the model’s generalizability and robustness in real-world clinical applications.

RL-Cervix.Net successfully addresses the research objectives by leveraging reinforcement learning to refine feature localization, outperforming existing SOTA methods, and demonstrating strong generalization across datasets. Its exceptional accuracy, interpretability, and potential for clinical integration position it as a significant advancement in AI-driven cervical cancer diagnostics, offering the promise of improved early detection and better patient outcomes.

## 7. Conclusions

This study introduces RL-Cervix.Net, a hybrid model combining a ResNet-50-based CNN with a reinforcement learning module for improved cervical cancer detection. RL-Cervix.Net achieves a remarkable 99.98% classification accuracy, surpassing existing models by refining feature localization through dynamic reward functions. Validated on extensive public datasets, the model demonstrates robustness and practical applicability, making it highly valuable for clinical use. RL-Cervix.Net significantly enhances diagnostic accuracy, interpretability, and reliability, reducing false positives and negatives—crucial for dependable medical diagnostics and better patient outcomes. This innovative approach highlights the potential of integrating RL with CNNs in medical diagnostics. The integration of reinforcement learning in RL-Cervix.Net represents a significant advancement, addressing key challenges of variability and interpretability in cervical cancer diagnostics. Future work will extend this approach to other medical domains to validate its robustness and versatility. For future research, we suggest to validate this model with larger datasets, explore applications for other cancers, and optimize computational efficiency for real-world deployment. RL-Cervix.Net represents a significant advancement in AI-driven medical diagnostics, promising improved early cancer detection and patient outcomes.

## Figures and Tables

**Table 1:** Summary of the important approaches discussed in the related work.

Method/Model	Key Characteristics	Dataset(s)	Computational Complexity
RL-CancerNet [19]	Reinforcement learning with EfficientNetV2; meta-learning ensemble	HErlev, Mendeley, SIPaKMeD	Moderate due to meta-learning
CACCD-GOADL [20]	Enhanced MobileNetv3; Gazelle Optimization Algorithm; SELM for classification	HErlev	Moderate
EnsembleCAM [21]	Combines five CAM techniques for enhanced interpretability	SIPaKMeD	Low to Moderate
CNN with SIPaKMeD [22]	CNN with four convolutional layers for five-class cervical cell classification	SIPaKMeD	Low
OPSCCnet [23]	Feature Pyramid Network; ResNet-18; tumor segmentation and classification	H&E Slides (906 patients)	High
CompactVGG [24]	Lightweight CNN for cervical cancer diagnosis	HErlev, SIPaKMeD	Low
DenseNet201 with SIPaKMeD [25]	Deep CNN model; highly accurate feature extraction	SIPaKMeD	High
SOD-GAN + F-SAE [26]	Small-Object Detection GAN; Fine-tuned Stacked Autoencoder	Custom dataset	High
CNN with VGG19 [27]	Transfer learning with VGG19 for colposcopy image classification	Colposcopy images	Moderate

**Table 2:** Descriptions of three well-known open-access datasets.

HErlev Dataset			Mendeley Dataset			SipaKMeD Dataset		
Category Name	Cell Category	Property	Category Name	Cell Category	Property	Category Name	Cell Category	Property
Intermediate squamous epithelial	70	Normal	Negative for intraepithelial malignancy	613	Normal	Parabasal	787	Normal
Superficial squamous epithelial	74		High squamous intraepithelial	113	Abnormal	Superficial-intermediate	813	
Columnar epithelial	98		Low grade squamous intraepithelial lesion	163		Dyskeratotic	813	Abnormal
Severe squamous non-keratinizing dysplasia	197	Abnormal	Squamous cell carcinoma	74		Koilocytotic	825	
Moderate squamous non-keratinizing dysplasia	146					Metaplastic	793	Benign
Mild squamous non-keratinizing dysplasia	182							
Squamous cell carcinoma in situ intermediate	150							
Total images	917		Total images	963		Total images	4049	

**Table 3:** Compares various models used for cervical cancer diagnosis, evaluated on four key metrics: accuracy, precision, recall, and F1 score. (HErlev, Mendeley, and SipaKMeD datasets).

Models	Accuracy	Precision	Recall	F1
ResNet50 [44]	98.89	99.32	98.84	99.78
Xcepsion [45]	97.87	97.18	97.99	97.85
EfficientNetV1 [46]	99.17	98.39	98.15	98.39
VGG 16 [47]	96.28	95.68	96.17	95.99
MobileNetV2 [48]	98.56	98.28	99.04	99.36
Inception [35]	97.23	97.10	96.74	97.14
ShuffleNet [49]	85.29	87.10	85.88	86.11
DenseNet 121 [50]	98.66	98.87	99.02	98.74
RL-Cervix.Net (ours)	99.98	99.92	99.89	99.90

**Table 4:** Presents a comparative analysis of various SOTA models for cervical cancer diagnosis, evaluated on four key performance metrics: accuracy, precision, recall, and F1 score. (HErlev, Mendeley, and SipaKMeD datasets).

Models	Accuracy	Precision	Recall	F1
RL-CancerNet [19]	99.70	99.36	99.90	99.73
CACCD-GOADL [20]	99.38	96.72	97.45	97.04
CNN -based [22]	97.88	97.64	97.87	98.17
MULTP [28]	96.54	96.87	96.15	96.93
SOD-GAN [29]	98.56	98.28	99.04	99.36
AttFPN [26]	99.17	98.81	99.27	99.30
RL-Cervix.Net (ours)	99.92	99.78	99.99	99.95

**Table 5:** The discussion substantiates the novelty of RL-Cervix.Net.

Model	Architectural Innovation	Datasets Used	Accuracy (%)	Precision (%)	Recall (%)	F1-Score (%)
RL-CancerNet [19]	Reinforcement learning integrated with EfficientNetV2	HErlev, SIPaKMeD	99.70	99.36	99.90	99.73
CACCD-GOADL [20]	Gazelle Optimization Algorithm with Deep Learning	HErlev, Mendeley	99.38	96.72	97.45	97.04
CNN-based [22]	Convolutional Neural Network with four convolutional layers	SIPaKMeD	97.88	97.64	97.87	98.17
AttFPN [26]	Attention-guided Feature Pyramid Network	SIPaKMeD, Mendeley	99.17	98.81	99.27	99.30
RL-Cervix.Net (ours)	ResNet-50 backbone with dynamic feature refinement via reinforcement learning and auxiliary attention mechanisms	HErlev, Mendeley, SIPaKMeD	99.98	99.92	99.99	99.95

## Data Availability

All used dataset are available online which open access.
